# Extranodal nasal–orbital communicating lesions NK/T cell lymphoma with ocular symptoms as the initial manifestation misdiagnosed as sinusitis and orbital cellulitis: a case report and literature review

**DOI:** 10.3389/fonc.2026.1732788

**Published:** 2026-04-01

**Authors:** Huijuan Li, Jie Liu, Lei Liu, Mingchang Guo, Shudong Tao, Dong Yang, Chaohui Yan

**Affiliations:** 1 Central Hospital, Tianjin University/Tianjin Third Central Hospital, Tianjin, China; 2 Tianjin Key Laboratory of Extracorporeal Life Support for Critical Diseases, Tianjin, China; 3 Tianjin Artificial Cell Engineering Technology Research Center, Tianjin, China; 4 Tianjin Institute of Hepatobiliary Disease, Tianjin, China

**Keywords:** Epstein-Barr virus, extranodal NK/T cell lymphoma, nasal-orbital region, orbital cellulitis, sinusitis

## Abstract

**Background:**

Extranodal natural killer/T cell lymphoma (ENKTL) is a non-Hodgkin lymphoma (NHL) with extranodal presentation.

**Case presentation:**

This report presents a rare case of ENKTL with ocular symptoms as the initial manifestation. Nasal endoscopy was normal. Magnetic resonance imaging (MRI) and sinus computed tomography (CT) scan revealed no evidence of mass or lymphadenopathy. The first hospitalization was misdiagnosed as sinusitis. Ophthalmic examination showed no significant decrease in vision. On 15 April 2025, a functional endoscopic sinus surgery (FESS) was performed. However, the patient’s condition worsened, leading to a second hospitalization. Postoperative CT shows changes in the area of the right inferior rectus muscle below the eyeball compared with preoperative CT, and the initial CT also showed a suspicious space-occupying lesion in the right orbital inferior orbital fissure region. Imaging examinations suggested orbital cellulitis. The first histopathological examination of the local mucosa did not provide a definitive tumor diagnosis. The second pathological examination was conducted. The patient lost vision in the right eye before the second set of pathological results came out. Based on examination results, the blindness may be caused by compression of the optic nerve due to swelling of the orbital tissues. A second surgery was performed urgently to restore the patient’s vision as soon as possible. Samples from six sites were sent for a third histopathological examination, and combined with the detection of Epstein–Barr virus (EBV), ENKTL was revealed, as in the second examination. The patient received gemcitabine/oxaliplatin (GemOx) chemotherapy. After five rounds of chemotherapy, he remains in remission, with no evidence that the lymphoma has recurred.

**Conclusions:**

It is relatively rare for ENKTL to involve intraocular or ocular adnexal tissues. The diagnosis is particularly challenging when patients present with facial swelling and periocular edema as the initial symptoms, especially when multiple CT and MRI examinations suggest the possibility of inflammatory lesions. When visiting our hospital, this patient presented with diplopia as the initial ophthalmologic complaint and ended up losing vision in the right eye, which was another “take-away” lesson of this case.

## 1 Introduction

Extranodal natural killer/T cell lymphoma (ENKTL) is an aggressive type of non-Hodgkin lymphoma (NHL) with a predilection for Asian and South American populations (1). ENKTL can be clinically divided into nasal and non-nasal subtypes (1). Non-nasal/extranasal can be in any location; typically, affected areas are the skin (most commonly), gastrointestinal tract, salivary glands, lungs, and testes (2). ENKTL will have the qualifier “nasal-type” dropped from its name in WHO-HAEM5 in accordance with the recognized presentation of this disease at various extranodal sites, as many extranodal non-nasal cases were well recognized, and dismal outcomes regarding topography are yet to be addressed (3–5) Primary orbital symptoms without nasal symptoms on presentation are infrequent, with only few cases in the literature (6–17). Cases with a dismal outcome of blindness are even rarer (9).

The periorbit is a highly resistant barrier to invasion, but once the tumor has passed through it, no further barriers prevent orbital content infiltration. Sinonasal benign and malignant tumors invading the orbit are rare and challenging to manage (18). Computed tomography (CT) of the paranasal sinuses is paramount for identifying orbital bone erosion or reabsorption and for assessing enlargement of fissures and foramina. Magnetic resonance imaging (MRI) is superior for analyzing orbital soft tissues and distinguishing inflammatory secretions from tumors.

Early diagnosis of ENKTL is challenging and is often delayed because it is misdiagnosed as other diseases, leading to treatment with antibiotics and antifungal drugs. Clinically, the primary differential diagnoses include chronic sinusitis or rhinitis, as well as infectious diseases such as nasal sclerosing disease, facial cellulitis, and deep fungal infections (19–21).

**Figure 1:**
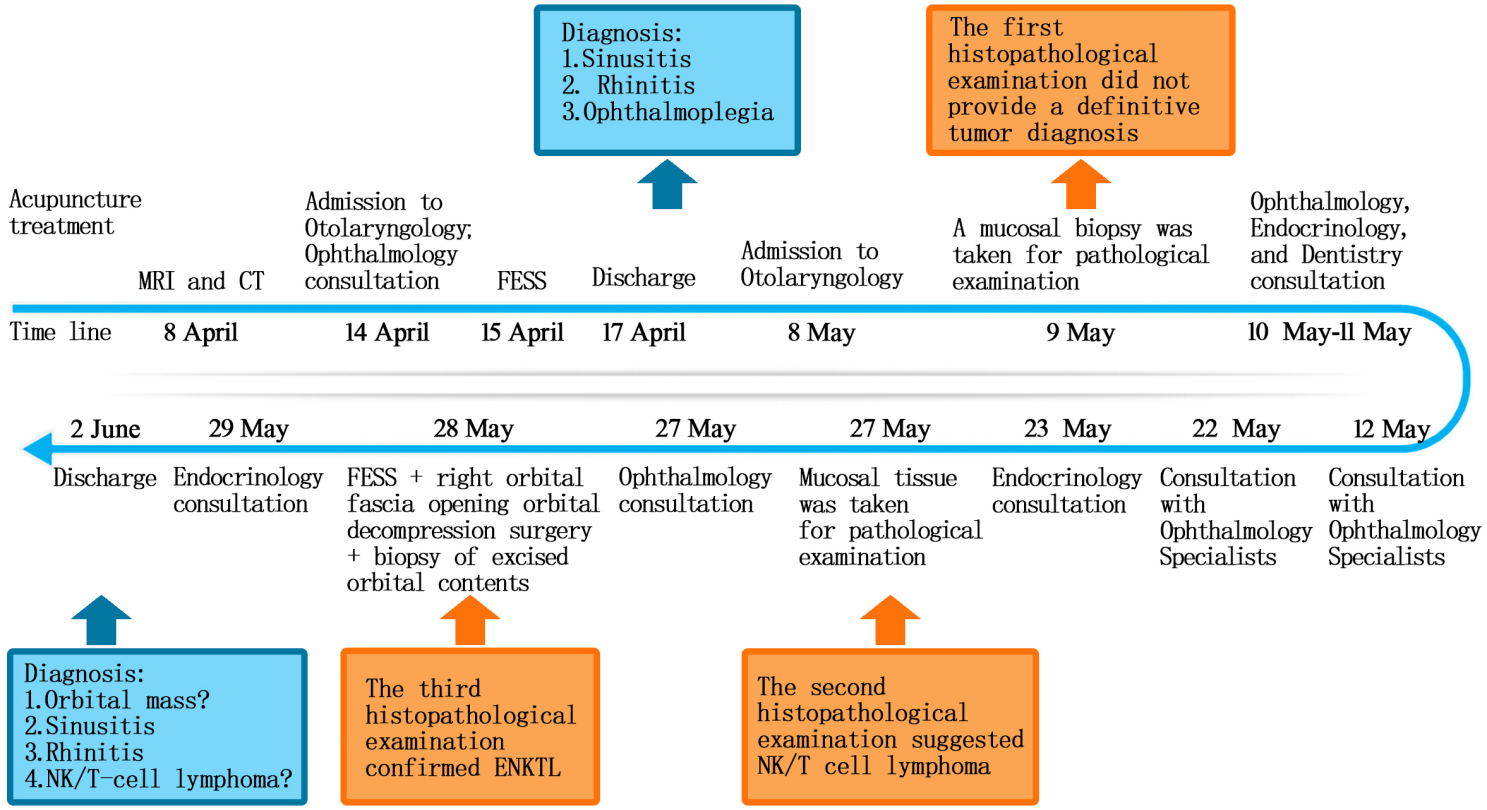
Timeline graphic illustrating the diagnostic and treatment process.

In this report, we present a rare case of ENKTL with orbital symptoms as the initial manifestation. The timeline is shown in **Figure 1**. Its nasal–orbital communicating lesions pose unique clinical challenges compared to isolated orbital diseases (22). Initially, it was misdiagnosed as sinusitis and orbital cellulitis, especially given multiple CT and MRI examinations suggesting the possibility of inflammatory lesions. When visiting our hospital, the patient presented with diplopia as the initial ophthalmologic complaint and ended up losing vision in the right eye, which was another “take-away” lesson of this case. Fortunately, we gave the correct diagnosis afterwards and saved the patient’s life, although there was permanent damage to the right eye and irreversible loss of vision. This case uses the gemcitabine/oxaliplatin (GemOx) chemotherapy regimen to provide clinicians with appropriate guidance.

## 2 Case presentation

### 2.1 Initial presentation

A 60-year-old man presented with a more than half-month history of swelling and pain in the right eye, accompanied by swelling, numbness, and pain on the right side of the face. The patient and the family members had no family medical history of tumors. The patient has had hypertension for 7–8 years and diabetes for 7–8 years. Acupuncture treatment was received at an external hospital. During the treatment process, the swelling, pain, and numbness on the right side of the face improved, but symptoms of diplopia appeared. Before visiting our otorhinolaryngology department, the patient had consulted an ophthalmology hospital and our neurology department, where sinusitis was considered, and surgery by the otorhinolaryngology department was recommended first. Therefore, the patient consulted our otorhinolaryngology department and was admitted with a diagnosis of “maxillary sinusitis (right)”.

### 2.2 The first hospitalization and misdiagnosis

**Figure 2:**
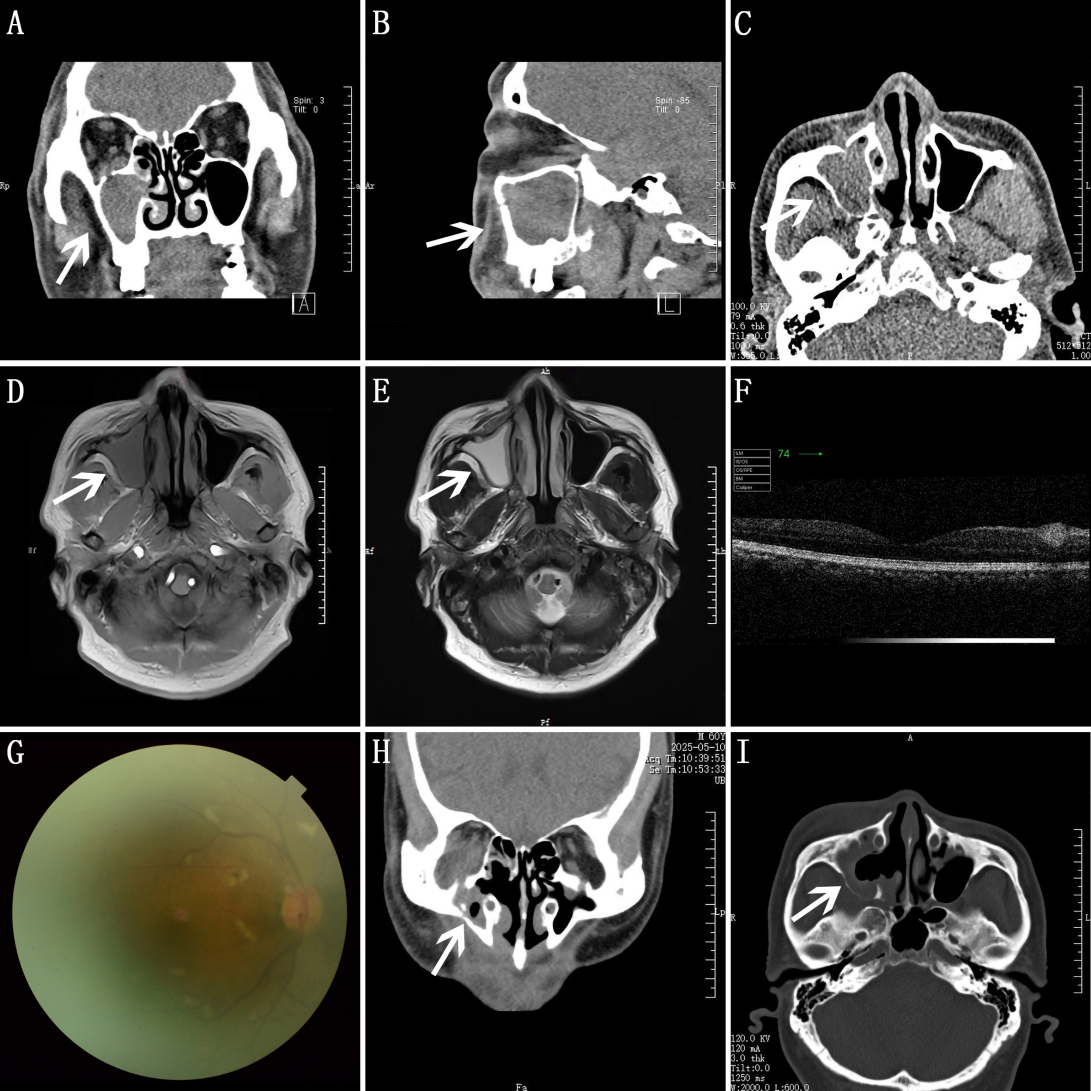
**(A–C)** Sinus CT findings before operation shows a slightly high-density shadow in the right maxillary sinus, with no obvious bone defect. **(A)** The coronal view. **(B)** The sagittal view. **(C)** The axial view. **(D, E)** Head MRI. **(D)** A short T1 signal shadow in the right maxillary sinus. **(E)** A long T2 signal shadow in the right maxillary sinus. **(F)** Optical coherence tomography of the right eye during the patient’s first hospitalization before blindness. **(G)** Fundus photography of the right eye during the patient’s first hospitalization before blindness. **(H, I)** Sinus CT findings after operation. **(H)** The coronal view. **(I)** The axial view.

Outpatient sinus CT scans (**Figures 2A–C**) and head MRI (8 April 2025) (**Figures 2D, E**) revealed an inflammatory lesion of the right maxillary sinus. The first hospitalization (14 April 2025 to 17 April 2025) was misdiagnosed as sinusitis. Laboratory tests were completed before surgery. Ophthalmology consultation considered that the symptoms may be caused by sinusitis. Ophthalmic examination showed no significant decrease in vision (**Supplementary Table 1**). Eyelid edema was observed, and downward eye movement was limited. Synoptophore examination suggested paralysis of the right inferior rectus muscle, and a follow-up synoptophore examination is recommended after treatment. Ocular B-scan ultrasound indicated vitreous opacities in both eyes. There were scattered, clustered medium-to-high reflectivity areas in the inner retina of both eyes (cotton-wool spots), with small cyst-like changes visible inside (**Figure 2F**). Fundus photography of the right eye showed a clear disk margin (**Figure 2G**). There were no surgical contraindications. A functional endoscopic sinus surgery (FESS) was performed on 15 April 2025. Intraoperative findings (**Supplementary Video**): Nasal endoscopy revealed there was no mass in the right nasal cavity. The mucosa around the ostium of the right maxillary sinus was swollen. After opening the sinus ostium, pale yellow, slightly purulent fluid flowed out. The maxillary sinus was irrigated until all secretions inside the sinus cavity were cleared, and the sinus mucosa was found to be smooth. The patient remained in good condition after the operation. The facial swelling and pain symptoms experienced significant relief at two postoperative days (PODs), but diplopia still existed. The patient and family were informed and advised to follow up at the Ophthalmology Hospital, and the patient was discharged.

### 2.3 Outpatient and emergency treatment

On POD 4, swelling around the right eye appeared; the patient returned to our outpatient clinic. Nasal endoscopy examination showed that the surgical area was recovering well; thus, the possibility of aggravated inflammation was considered. There was a slight improvement with antibiotic medication.

On POD 11, the patient returned to our emergency department, considering a progressive condition: acute sinusitis? orbital cellulitis? The patient was managed with antibiotics and hormonal support. Twelve days later, the swelling around the right eye persisted. Additionally, ulcers appeared around the hard palate and gingiva, which led to a second hospitalization (8 May 2025 to 2 June 2025).

### 2.4 The second hospitalization and confirmation

On 9 May 2025, the right maxillary sinus was flushed through the natural ostium. Mucosal swelling and dry scabs were observed within the maxillary sinus. The sinus cavity secretions were flushed thoroughly, and a mucosal biopsy was taken for pathological examination. The first histopathological examination did not provide a definitive tumor diagnosis (**Supplementary Figures 1A, B**). Immunohistochemistry was negative for CK (**Supplementary Figure 1C**); positive for LCA (**Supplementary Figure 1D**), and VIM (**Supplementary Figure 1E**); and focally positive for Ki67 (**Supplementary Figure 1F**). Bacterial culture results for secretion were negative.

**Figure 3:**
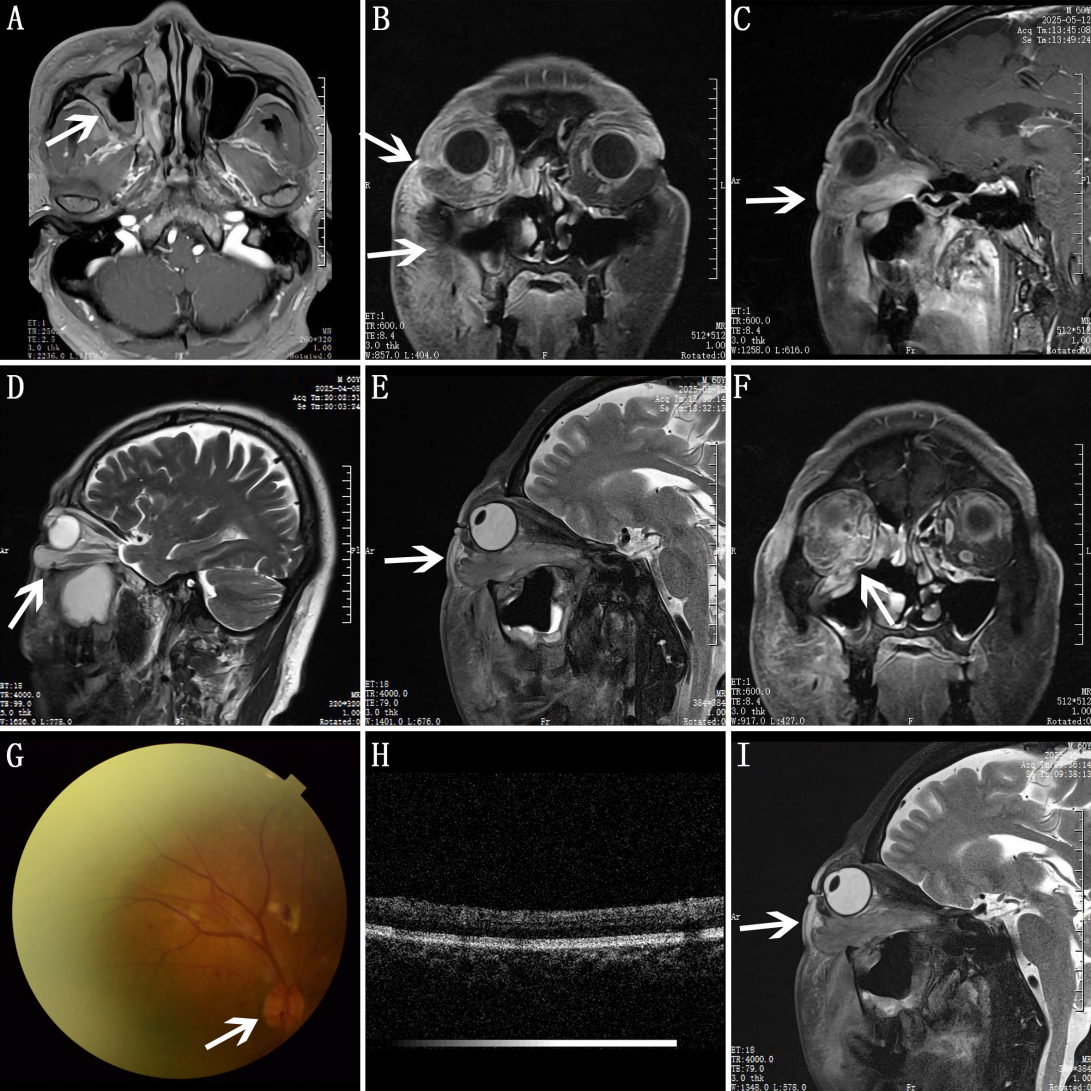
**(A–C)** MRI reveals that there was increased signal in the fat space behind the lower part of the right eye, accompanied by swelling of the right inferior rectus muscle. Right-sided proptosis was present, suggesting an inflammatory lesion. **(A)** Normal changes during postoperative recovery. **(B)** The coronal view. **(C)** The sagittal view. **(D)** The inferior rectus muscle region of orbital MRI before the FESS surgery. **(E)** The inferior rectus muscle region of orbital MRI after the FESS surgery. **(F)** The enhanced MRI showed bone defects. **(G)** Fundus photography of the right eye during the patient’s second hospitalization after blindness. **(H)** Optical coherence tomography of the right eye during the patient’s second hospitalization after blindness. **(I)** The inferior rectus region of orbital MRI after blindness.

Sinus CT scan with 3D reconstruction (**Figures 2H, I**) and contrast-enhanced orbital MRI (**Figures 3A–C**) revealed that the sinuses appear normal. There was an increased signal in the fat space behind the lower part of the right eye, accompanied by swelling of the right inferior rectus muscle. Right-sided proptosis was present, which suggests an inflammatory lesion.

Ophthalmic examination revealed obvious congestion and edema of the right eyelid, with no significant tenderness (**Supplementary Table 1**). The conjunctiva also showed congestion and edema, and the limitation of eye movement was more severe than last time. Ophthalmology consultation diagnosed a space-occupying lesion in the right orbit (inflammatory? malignant tumor)?. A blood smear was recommended to check for abnormal cells. A local tissue biopsy should be conducted to confirm the pathological diagnosis. Color Doppler flow imaging (CDFI) should be used to observe the relationship between the lesion under the right orbit and the inferior rectus muscle. If the biopsy pathology result is negative, tissue from the intraorbital lesion or the oral ulcer region should be obtained for further confirmation. A CDFI of cervical lymph nodes (LNs) should be performed to evaluate for any abnormalities.

Postoperative CT (**Supplementary Figures 2A, B**) shows changes in the area of the right inferior rectus muscle below the eyeball compared with before surgery, and the initial CT (**Supplementary Figures 2C, D**) also showed a suspicious space-occupying change in the right orbital inferior orbital fissure region. Postoperative orbital MRI suggests inflammatory changes inside the orbit, and a comparison of the two orbital MRIs shows significant worsening swelling in the inferior rectus muscle region (**Figures 3D, E**). Considering that the patient’s response to antibiotics and hormonal support treatments was not obvious, and in combination with the ophthalmologist’s consultation, the second pathological examination was conducted by taking mucosal tissue from the top wall of the right maxillary sinus near the orbital floor, where enhanced MRI showed bone defects (**Figure 3F**).

On 27 May 2025, the patient felt severe swelling and pain in the right eye and the right side of the face. The patient could not see with the right eye. During the ophthalmic examination, the patient’s vision had deteriorated to hand motion only. The right eyelid was noticeably swollen, making it difficult to open the eye. There was conjunctival congestion and edema. The pupillary light reflex showed that the pupil diameter had reached 5 mm (**Supplementary Table 1**), and the light reflex was very sluggish. Eye movement was still restricted. An urgent fundus examination (**Figure 3G**) and optical coherence tomography (OCT) of the fundus (**Figure 3H**) revealed that the color of the optic nerve was basically normal, with no signs of optic nerve edema, and the vascular course was generally normal. After blindness, further refinement of orbital MRI (**Figure 3I**) showed that there was no significant change in the swelling of the inferior rectus region before and after blindness. Inflammatory lesions were still indicated. The blindness may be caused by compression of the optic nerve due to swelling of orbital tissues. On 28 May 2025, right-sided FESS via nasal endoscopy + right orbital fascia opening, orbital decompression surgery + biopsy of excised orbital contents was performed. Samplings from six sites (right maxillary sinus, inside the right orbit, right infraorbital, inferior part of the right orbit, inferior bone of the right orbit, and right orbital bone) were sent for the third histopathological examination. Next-generation sequencing (NGS) detected Epstein–Barr virus (EBV) infection, which provided direction for diagnosis.

**Figure 4:**
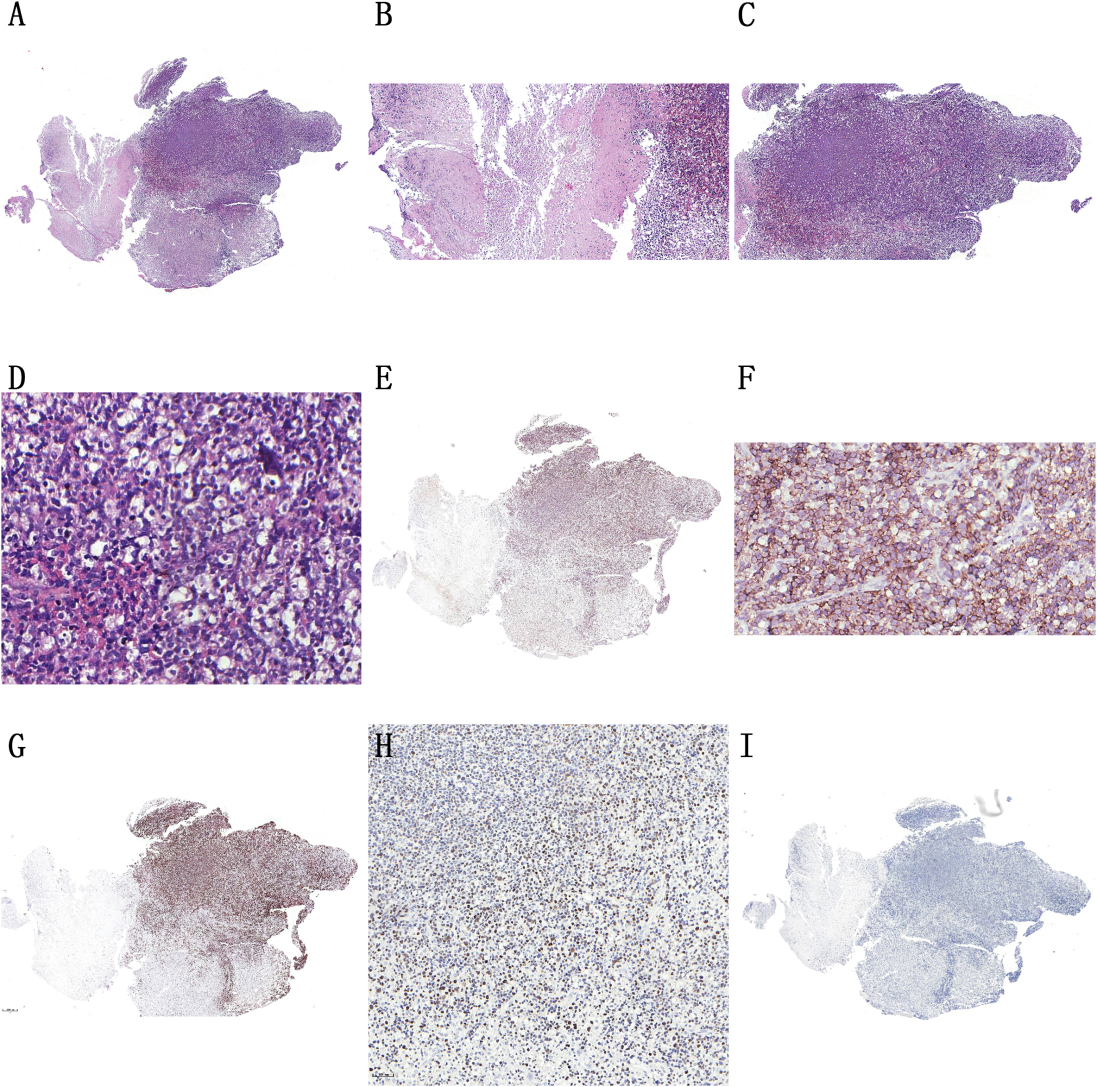
Representative histopathologic and immunohistochemical findings in a patient with ENKTL. **(A)** H&E stain, ×40: No blood vessels or destruction observed in the sterile mass. **(B)** H&E stain, ×100: Necrosis components. **(C)** H&E stain, ×100: Diffuse infiltration of lymphocytes. **(D)** H&E stain, ×400: Small to medium-sized tumor cells. **(E)** CD3, ×40. **(F)** CD45RO, ×400. **(G)** Ki67, ×40. **(H)** EBER, ×200. **(I)** CD21, ×40.

On 31 May 2025, the second histopathological examination suggested NK/T cell lymphoma (**Figures 4A–D**); the mucosal tissue sample from the roof of the right maxillary sinus near the orbital floor was limited; further complete relevant examinations were needed for a definitive diagnosis. Immunohistochemistry for the mucosal tissue sample was positive for CD3 (**Figure 4E**), CD45Ro (**Figure 4F**), Ki67 (**Figure 4G**), and EBV-encoded RNA (EBER) (**Figure 4H**), and negative for CK, HMB45, CD20, CD79a, Bcl-2, Bcl-6, CD5, CyclinD1, CD10, CD21 (**Figure 4I**), and SOX-11. Based on CD21 negativity and Ki67 positivity, malignancy was confirmed. Examining the cell origin, T-cell markers CD3 and CD45RO were positive, while the B-cell marker CD20 was negative, indicating that it is of T-cell origin. Since NK/T cells are most numerous in the nasal cavity, NK markers were planned, but CD56 was out of stock; thus, it was unfortunately not stained. However, EBER hybridization was positive. Based on CD20(−) and CD10(−), diffuse large B-cell lymphoma and MALT lymphoma were excluded. Based on Bcl-2(−) and Bcl-6(−), follicular lymphoma was excluded. Based on CyclinD1(−) and SOX-11(−), mantle cell lymphoma was excluded. Based on CK(−), nasopharyngeal carcinoma was excluded. Based on HMB45(−), malignant melanoma was excluded. Therefore, a diagnosis of NK/T cell lymphoma was made.

On 2 June 2025, the swelling and pain on the right side of the face and eye had lessened, but there was no light perception in the right eye. Blindness is associated with tumor infiltration of the optic nerve. Upon contacting the Ophthalmology Hospital, it was recommended that the patient be transferred there for further diagnosis and treatment. After informing the patient and family about the current condition and treatment plan, the patient provided informed consent to continue the diagnosis and treatment at the Ophthalmology Hospital and was discharged.

On 5 June 2025, the third histopathological examination confirmed extranodal NK/T cell lymphoma (**Supplementary Figures 3A, B**). In specimen #5, a large number of atypical cells were observed between the trabeculae. Further testing is recommended. Immunohistochemistry results for specimen #5 showed the following: it was positive for EBER (**Supplementary Figure 3C**), LCA (**Supplementary Figure 3D**), VIM (**Supplementary Figure 3E**), CD99 (**Supplementary Figure 3F**), Ki67 (8%) (**Supplementary Figure 3G**), and CD45Ro, and negative for CK (**Supplementary Figure 3H**), P53, CD34, CD3, CD20 (**Supplementary Figure 3I**), and CD79a. The CD3 result is negative, which may be due to procedures during specimen processing and decalcification of this bone specimen, leading to the potential loss of some antigens. For this reason, even though the Ki-67 index is not very high, the initial diagnosis still considers the tumor to be malignant. Diffuse positivity for LCA indicates a lymphocyte origin. Combined with CD20 negativity, a B-cell origin is excluded. Strong positivity for CD45RO still suggests a T-cell origin. EBER hybridization is positive, supporting a consideration of ENKTL.

On 12 June, combining immunohistochemical staining and pathogen metagenomic testing, the pathological results (right orbit and maxillary sinus) from the Ophthalmology Hospital were consistent with T-cell lymphoma (tending towards extranodal NK/T cell lymphoma), accompanied by extensive tumor cell necrosis, with tumor cells invading nerves and adipose tissue.

### 2.5 The third hospitalization and complications

On the morning of 11 June at 6:18 a.m., the patient became unconscious suddenly and responded when called, and body temperature was measured at 39.4°C. According to laboratory tests, infection as a cause cannot be ruled out. After antibiotics and hormonal support, the patient’s consciousness recovered, but the body temperature remained high. By using a higher-generation antibiotics, the body temperature peak decreased compared to before. The patient had a high fever, which was caused by both infection and lymphoma. Experts from the lymphoma department of an external hospital considered that the patient had pancytopenia and recommended anti-infection treatment followed by radiotherapy.

A head CT scan showed no definite abnormalities. Neck CT scan revealed protrusion of the right eyeball, increased density in the fat spaces beneath the right eye and behind the globe, and thickening of the right inferior rectus and medial rectus muscles. Increased subcutaneous fat density was observed in the right periorbital and maxillofacial regions, with local gas shadows visible. Multiple significantly enlarged LNs are seen in right cervical regions I and II. Consider the possibility of soft tissue lymphoma of the right periorbital and maxillofacial areas with infectious cellulitis, along with multiple enlarged cervical LNs. Chest and abdominal CT scan with and without contrast suggests bilateral pulmonary inflammatory lesions and right pleural effusion. Multiple hepatic cavernous hemangiomas were presented, bilateral adrenal adenomas were considered, and a mass was detected in the lower pole of the right kidney (possibly renal cancer? lymphoma involvement?). Lymphoma was accompanied by right periocular and maxillofacial subcutaneous cellulitis; both anti-infection and anti-tumor treatments should be considered.

The patient is highly suspected of having hemophagocytic syndrome (HPS): fever lasting for more than 1 week, splenomegaly, cytopenia [WBC 2.79×10^9^/L (3.5–9.5), Hb 92 g/L (130–175), and PLT 88×10^9^/L (125–350)], fasting triglycerides 2.73 mmol/L (0.0–1.7), lactate dehydrogenase 444 U/L (120.0–250.0), ferritin 956.6 ng/mL (30–400), high-density lipoprotein 0.88 (1.16–1.42), and EBV infection. The patient was discharged after the symptoms improved.

The clinical subtype is ENKTL nasal according to the WHO classification system (23). The lymphoma is staged at IVB according to the final diagnosis from the Cancer Institute and Hospital. Gemcitabine is a novel nucleoside analog that primarily acts on S-phase cells, inhibiting DNA synthesis and thereby inducing apoptosis. This mechanism endows gemcitabine with broad-spectrum anti-tumor activity (24). Furthermore, the mechanisms of gemcitabine and oxaliplatin are distinct, and their combination produces a synergistic effect. Therefore, combination therapy is more effective compared with monotherapy (25). According to the patient’s age and performance, the patient received GemOx chemotherapy, finally. The patient experienced side effects from chemotherapy, including weakness, loss of appetite, and bone marrow suppression. The patient underwent five rounds of chemotherapy after a precise diagnosis. He remains in remission, with no evidence that the lymphoma has recurred. The patient is now able to care for himself.

## 3 Discussion

This case highlights the significant diagnostic challenge when ENKTL presents primarily with orbital symptoms, especially when CT and MRI suggest only the possibility of inflammatory lesions and nasal endoscopy is normal. Its nasal–orbital communicating lesions pose unique clinical challenges compared to isolated orbital diseases. When visiting our hospital, the patient presented with diplopia as the initial ophthalmologic complaint and ended up with permanent damage to the right eye and irreversible loss of vision. Cases with a dismal outcome of blindness are rarely reported.

ENKTL is a life-threatening disease characterized by excessive stimulation of the immune system, leading to systemic inflammation and multiple organ failure. It is rare and difficult to diagnose, with no specific imaging indicators (26). Tumor tissue is mixed with infected mucosa and dried scabs, making accurate sampling challenging. Multiple biopsies are often required to confirm the diagnosis, and the disease progresses rapidly with a poor prognosis in advanced stages. Pathology from multiple sites, with sufficiently large and repeated sampling, may improve the accuracy of pathological diagnosis, which is essential to determine the nature and origin of the tumor.

When visiting our hospital, the patient presented with diplopia as the initial ophthalmologic complaint. After examinations, the ophthalmology consultation diagnosed (14 April) the following: (1) extraocular muscle paralysis (right inferior rectus) and (2) hypertensive retinopathy in both eyes. A second round of eye examinations (11 May-12 May) was performed during the second hospitalization. Postoperative CT shows changes in the area of the right inferior rectus muscle below the eyeball compared to before surgery, and the initial CT also shows a suspicious mass in the right orbital floor region, which was an important “take-away” lesson of this case retrospective. When the patient could not see with the right eye (27 May), a series of examinations was performed to identify the cause of blindness. Based on all examination results (27 May), blindness may be caused by compression of the optic nerve due to swelling of orbital tissues, after thoroughly discussing the details with the patient and family. Right-sided orbital fascia opening, orbital decompression surgery was done urgently, aiming to restore the patient’s vision as soon as possible. After the surgery, there was no light perception in the right eye. Combined with the results of the Ophthalmology Hospital examinations, blindness is associated with tumor infiltration of the optic nerve. Although there was permanent damage to the right eye and irreversible loss of vision, we gave the correct diagnosis promptly and saved the patient’s life.

The patient was very grateful to all the medical staff at the hospital for their treatment and care, sincerely thanking the doctors and nurses for their professionalism and patience, which enabled the patient to receive subsequent treatment smoothly and ignited hope for recovery. Over the past 20 years, 11 patients diagnosed with orbital NKTCL underwent pathological examination at Beijing Tongren Hospital, Capital Medical University. At the time of initial diagnosis, all patients were misdiagnosed as having inflammatory pseudotumor (six cases), orbital cellulitis (three cases), or conjunctivitis (two cases) (17). This case emphasizes the differentiation from inflammatory pseudotumor and orbital cellulitis. Inflammatory pseudotumor minimally affected the optic nerve, vision impairment is mild, and the effect of steroids is significant (17). Orbital cellulitis (27) often has a sudden onset (from a few hours to a few days), with apparent tenderness, is commonly accompanied by fever (a key point in the differential diagnosis for this case), and rarely involves nasal symptoms; bone destruction is uncommon (an important lesson from this case). Therefore, the recommendation for this case is that, if the lymphoma does not respond to conventional therapy (steroids and antibiotics) and CT and MRI results show no significant findings, the timely use of EBV and EBER is paramount.

ENKTL is an EBV-associated extranodal lymphoma derived from NK or T cells (28). There is a strong association between EBV and ENKTL. EBV occurs in tumor cells as a clonal episome (29). In this case report, NGS detection of EBV guided the disease diagnosis. The methods for detecting EBV infection in this case include NGS and an EBER test on the tissue. NGS can help us detect infections caused by bacteria, viruses, or parasites (30). Its application of EBV detection not only allows identification of the virus’ presence but also enables analysis of its whole genome, distinguishing EBV types and providing detailed data on viral mutations and drug resistance. The use of molecular diagnostic techniques in EBV detection offers powerful tools to the medical community. However, high costs and complex operational requirements limit its widespread use in primary healthcare. EBER involves detecting EBV-encoded small RNAs in tissue samples. This result is often used to determine whether the disease is related to EBV infection and provides a reference for disease classification, treatment plan selection, and prognosis assessment. Analyzing the two detection methods from the perspective of specimen types, when a patient is suspected of having lymphoma but tissue samples cannot be obtained, collecting the patient’s blood sample for NGS testing is undoubtedly the most beneficial.

EBV is a ubiquitous, oncogenic virus that is associated with several different human malignancies as well as autoimmune disorders: infectious mononucleosis, EBV-induced lymphoproliferative disease, Burkitt lymphomas, Hodgkin lymphomas, diffuse large B-cell lymphomas, NK and T-cell lymphoproliferative diseases, nasopharyngeal carcinoma, gastric carcinoma, and autoimmune diseases associated with EBV (31). EBV screening can help prevent diseases early for those who have mosquito bite hypersensitivity (32), diabetes (33), schizophrenia (34), lung transplantation (35), primary immunodeficiencies (36), etc. A prospective study investigated whether EBV DNA in plasma samples could be useful for screening for early nasopharyngeal carcinoma in asymptomatic individuals (37). Diseases were detected significantly earlier, and outcomes were better, among participants identified through screening. A sample of venous blood was obtained, and EBV DNA in plasma was analyzed using real-time polymerase chain reaction. In participants with positive results, another blood sample was obtained approximately 4 weeks later. Participants with persistently positive results were referred for endoscopic examination and MRI of the nasopharynx. Forty-three months (median) after the initial screening, a second round of screening was conducted on the same population, using the same methods for individuals without cancer (38). Patients identified through screening have superior survival compared with those who do not undergo screening. EBV infects epithelial cells in the oropharynx, where it can replicate and subsequently infect B cells that traffic to the oral cavity (31). EBV DNA load testing in nasopharyngeal swabs can also be used for screening (39).

## 4 Conclusion

In patients suspected of HPS, glycated albumin has a higher reference value than glycated hemoglobin in the treatment of diabetes. During the third hospitalization, chest and abdominal CT scan with and without contrast suggests that there was a mass in the lower pole of the right kidney (possibly renal cancer? lymphoma involvement)?. These issues are all ones we need to pay attention to during the later stages of treatment.

The patient and the family members had no family history of genetic diseases. So why did he develop lymphoma? The difference lies in him having had hypertension for 7–8 years, diabetes for 7–8 years, and an EBV infection (discovered during this hospitalization). A comparative study published in *The Lancet* in 2017 reported (40): Disparities by province, age, and sex in site-specific cancer were attributable to 23 potentially modifiable risk factors in China. Diabetes and EBV are potentially modifiable risk factors for cancer. By establishing an EBV screening system, we can prevent the occurrence of diseases early.

We have some reflections from this case: (a) The patient exhibits “masked” symptoms such as facial swelling and periorbital edema. (b) Multiple imaging examinations suggest the possibility of inflammatory lesions. (c) The first histopathological findings did not provide a definitive tumor diagnosis. (d) There is facial and eye swelling without obvious congestion or ulceration. Patients with ENKTL often first present with symptoms of the ear, nose, and throat. (e) Because of the limitation of a short follow-up period, oncology management is still not fully developed. By increasing our understanding of ENKTL, we can make timely diagnoses and predict patient prognosis.

## Data Availability

The original contributions presented in the study are included in the article/**Supplementary Material**. Further inquiries can be directed to the corresponding authors.

## Supplementary material

Supplementary Figure 1 Representative histopathologic and immunohistochemical findings in a patient with ENKTL. **(A)** H&E stain, ×100: Squeezed blue-stained unstructured diffuse cells. **(B)** H&E stain, ×40: Squeezed blue-stained unstructured diffuse cells. **(C)** CK, ×40. **(D)** LCA, ×200. **(E)** Vim, ×100. **(F)** Ki67, ×100.

Supplementary Figure 2 **(A, B)** The area of the right inferior rectus muscle below the eyeball after the FESS surgery. **(A)** The coronal view. **(B)** The sagittal view. **(C, D)** The area of the right inferior rectus muscle below the eyeball before the FESS surgery. **(C)** The coronal view. **(D)** The sagittal view.

Supplementary Figure 3 Representative histopathologic and immunohistochemical findings in a patient with ENKTL. **(A)** Samplings from 6 sites (1. Inferior part of the right orbit 2. Right orbital bone 3. Inferior bone of the right orbit 4. Right infraorbital 5. Inside the right orbit 6. Right maxillary sinus). **(B)** H&E stain, ×200. **(C)** EBER ×200. **(D)** LCA, ×400. **(E)** VIM, ×400. **(F)** CD99, ×200. **(G)** Ki67, ×200. **(H)** CK, ×200. **(I)** CD20, × 200.

## References

[1] Tse E Zhao WL Xiong J Kwong YL . How we treat NK/T-cell lymphomas. J Hematol Oncol. (2022) 15:74. doi: 10.1186/s13045-022-01293-5, PMID: 35659326 PMC9164389

[2] Au WY Weisenburger DD Intragumtornchai T Nakamura S Kim WS Sng I . Clinical differences between nasal and extranasal natural killer/T-cell lymphoma: a study of 136 cases from the International Peripheral T-Cell Lymphoma Project. Blood. (2009) 113:3931–7. doi: 10.1182/blood-2008-10-185256, PMID: 19029440

[3] Isobe Y Aritaka N Sasaki M Oshimi K Sugimoto K . Spontaneous regression of natural killer cell lymphoma. J Clin Pathol. (2009) 62:647–50. doi: 10.1136/jcp.2008.062976, PMID: 19561234

[4] Alaggio R Amador C Anagnostopoulos I Attygalle AD Araujo IBO Berti E . The 5th edition of the world health organization classification of haematolymphoid tumours: lymphoid neoplasms. Leukemia. (2022) 36:1720–48. doi: 10.1038/s41375-022-01620-2, PMID: 35732829 PMC9214472

[5] Costa RO Pereira J Lage LAPC Baiocchi OCG . Extranodal NK-/T-cell lymphoma, nasal type: what advances have been made in the last decade? Front Oncol. (2023) 13:1175545. doi: 10.3389/fonc.2023.1175545, PMID: 37529691 PMC10388588

[6] Yang Y Luo Q He W Tang L . Primary ocular natural killer/T-cell lymphomas: clinicopathologic features and diagnosis. Ophthalmologica. (2007) 221:173–9. doi: 10.1159/000099297, PMID: 17440279

[7] Hon C Kwok AK Shek TW Chim JC Au WY . Vision-threatening complications of nasal T/NK lymphoma. Am J Ophthalmol. (2002) 134:406–10. doi: 10.1016/s0002-9394(02)01520-9, PMID: 12208253

[8] Marchino T Ibáñez N Prieto S Novelli S Szafranska J Mozos A . An aggressive primary orbital natural killer/T-cell lymphoma case: poor response to chemotherapy. Ophthalmic Plast Reconstr Surg. (2014) 30:e131–4. doi: 10.1097/IOP.0b013e3182a65026, PMID: 24317101

[9] Dai W Zhong M Shen W Zou K Bai CG . Natural killer T-cell lymphoma originating from the orbit. Chin Med J (Engl). (2012) 125:1677–80. 22800846

[10] Kuwabara H Tsuji M Yoshii Y Kakuno Y Akioka T Kotani T . Nasal-type NK/T cell lymphoma of the orbit with distant metastases. Hum Pathol. (2003) 34:290–2. doi: 10.1053/hupa.2003.33, PMID: 12673566

[11] Woog JJ Kim YD Yeatts RP Kim S Esmaeli B Kikkawa D . Natural killer/T-cell lymphoma with ocular and adnexal involvement. Ophthalmology. (2006) 113:140–7. doi: 10.1016/j.ophtha.2005.09.036, PMID: 16360212

[12] Ely A Evans J Sundstrom JM Malysz J Specht CS Wilkinson M . Orbital involvement in extranodal natural killer T cell lymphoma: an atypical case presentation and review of the literature. Orbit. (2012) 3:267–9. doi: 10.3109/01676830.2011.605506, PMID: 22681504

[13] Coupland SE Foss HD Assaf C Auw-Haedrich C Anastassiou G Anagnostopoulos I . T-cell and T/natural killer-cell lymphomas involving ocular and ocular adnexal tissues: a clinicopathologic, immunohistochemical, and molecular study of seven cases. Ophthalmology. (1999) 106:2109–20. doi: 10.1016/S0161-6420(99)90492-X, PMID: 10571346

[14] Qin W Yin Z Madge SN . Acute presentation of nasal-type natural killer/T-cell lymphoma of the orbit. Eur J Ophthalmol. (2009) 19:679–82. doi: 10.1177/112067210901900425, PMID: 19551687

[15] Lee J Kim WS Park YH Park SH Park KW Kang JH . Nasal-type NK/T cell lymphoma: clinical features and treatment outcome. Br J Cancer. (2005) 92:1226–30. doi: 10.1038/sj.bjc.6602502, PMID: 15798768 PMC2361983

[16] Termote K Dierickx D Verhoef G Jorissen M Tousseyn T Mombaerts I . Series of extranodal natural killer/T-cell lymphoma, nasal type, with periorbital involvement. Orbit. (2014) 33:245–51. doi: 10.3109/01676830.2014.902478, PMID: 24831171

[17] Li J Ren T Liu R Zhang H Wang N Guo Q . Orbital natural killer/T-cell lymphoma: a comprehensive case series and literature review. BMC Cancer. (2025) 25:372. doi: 10.1186/s12885-025-13761-5, PMID: 40022004 PMC11869575

[18] Castelnuovo P Lambertoni A Sileo G Valentini M Karligkiotis A Battaglia P . Critical review of multidisciplinary approaches for managing sinonasal tumors with orbital involvement. Acta Otorhinolaryngol Ital. (2021) 41:S76–89. doi: 10.14639/0392-100X-suppl.1-41-2021-08, PMID: 34060523 PMC8172109

[19] Sánchez-Romero C Paes de Almeida O Rendón Henao J Carlos R . Extranodal NK/T-cell lymphoma, nasal type in Guatemala: an 86-case series emphasizing clinical presentation and microscopic characteristics. Head Neck Pathol. (2019) 13:624–34. doi: 10.1007/s12105-019-01027-z, PMID: 30900209 PMC6854135

[20] Celentano A Mascolo M Cirillo N De Rosa G Mignogna MD . Delayed diagnosis of a nasal type lymphoma misdiagnosed as persistent sinusitis. J Adolesc Young Adult Oncol. (2017) 6:381–4. doi: 10.1089/jayao.2016.0050, PMID: 28061034

[21] Reategui Schwarz E Oikonomou KG Reynolds M Kim J Balmiki RL Sterling SA . Extranodal NK/T-cell lymphoma, nasal type, presenting as refractory Pseudomonas aeruginosa facial cellulitis. J Investig Med High Impact Case Rep. (2017) 5:2324709617716471. doi: 10.1177/2324709617716471, PMID: 28748192 PMC5507379

[22] Xie M Chen J You YY Su ZX Zhu XY Wang XH . Clinicopathological features of cranial-nasal-orbital communicating lesions and diagnostic indicators for differentiating benign and Malignant neoplasms. Int J Ophthalmol. (2025) 18:1357–68. doi: 10.18240/ijo.2025.07.20, PMID: 40688786 PMC12207302

[23] Naresh KN Medeiros LJ WHO Fifth Edition Classification Project . Introduction to the fifth edition of the world health organization classification of tumors of hematopoietic and lymphoid tissues. Mod Pathol. (2023) 36:100330. doi: 10.1016/j.modpat.2023.100330, PMID: 37716508

[24] Zhang Z Yu H Yao W Zhu N Miao R Liu Z . RRP9 promotes gemcitabine resistance in pancreatic cancer via activating AKT signaling pathway. Cell Commun Signal. (2022) 20:188. doi: 10.1186/s12964-022-00974-5, PMID: 36434608 PMC9700947

[25] Zhang X Li G Liu F Shi R Wen J Wu W . Analysis of the efficacy and prognostic factors of gemcitabine combined with oxaliplatin in non-Hodgkin lymphoma. Oncol Lett. (2025) 30:603. doi: 10.3892/ol.2025.15349, PMID: 41181627 PMC12576767

[26] de Leval L Gaulard P Dogan A . A practical approach to the modern diagnosis and classification of T- and NK-cell lymphomas. Blood. (2024) 144:1855–72. doi: 10.1182/blood.2023021786, PMID: 38728419 PMC11830980

[27] Luo J Tao R Liu CX Ma YJ Gu VY Li J . Ocular manifestations of patients with extranodal NK/T-cell lymphoma. Eye (Lond). (2025) 39:3243–8. doi: 10.1038/s41433-025-04052-1, PMID: 41057714 PMC12669774

[28] Jeon YK Kim H Park SO Choi HY Kim YA Park SS . Resistance to Fas-mediated apoptosis is restored by cycloheximide through the downregulation of cellular FLIPL in NK/T-cell lymphoma. Lab Invest. (2005) 85:874–84. doi: 10.1038/labinvest.3700291, PMID: 15924153

[29] Chan WL Hue SS Deng L Leong SM Chng WJ Ng SB . Extranodal NK/T-cell lymphoma: an update of the molecular characterization of the tumor and microenvironment, and its clinical implications. Lancet Reg Health West Pac. (2025) 62:101550. doi: 10.1016/j.lanwpc.2025.101550, PMID: 41132165 PMC12541150

[30] Ji H Zhan S Yang N Hou X Li J Zheng J . Clinical decision making impact of Next-Generation Sequencing in Pneumocystis jirovecii infection: infection characteristics and prognosis. Diagn Microbiol Infect Dis. (2026) 114:117175. doi: 10.1016/j.diagmicrobio.2025.117175, PMID: 41192287

[31] Damania B Kenney SC Raab-Traub N . Epstein-Barr virus: Biology and clinical disease. Cell. (2022) 185:3652–70. doi: 10.1016/j.cell.2022.08.026, PMID: 36113467 PMC9529843

[32] Higa M Atsumi E Hoshino H Yamashiro M Nakajima T Tomita M . Extranodal natural killer/T-cell lymphoma with lung involvement in a young adult with suspected mosquito bite hypersensitivity: A case report. Respir Investig. (2025) 63:872–6. doi: 10.1016/j.resinv.2025.07.002, PMID: 40639036

[33] Midorikawa S Mizukami H Kudoh K Takeuchi Y Sasaki T Kushibiki H . Diabetes can increase the prevalence of EBV infection and worsen the prognosis of nasopharyngeal carcinoma. Pathology. (2024) 56:65–74. doi: 10.1016/j.pathol.2023.09.013, PMID: 38071160

[34] Sighencea MG Trifu SC . Unravelling the viral hypothesis of schizophrenia: A comprehensive review of mechanisms and evidence. Int J Mol Sci. (2025) 26:7429. doi: 10.3390/ijms26157429 40806558 PMC12347704

[35] Ma T Qin J Deng Y . Intrapulmonary Epstein-Barr virus-associated smooth muscle tumor after bilateral lung transplantation: A case report. Transplant Proc. (2025) 57:1376–8. doi: 10.1016/j.transproceed.2025.07.017, PMID: 40796387

[36] van den Brand M Rásó-Barnett L Gasljevic G Balague O Laurent C Ponzoni M . Atypical lymphoproliferations associated with germline genetic variants: a report of the 2024 EA4HP/SH lymphoma workshop. Virchows Arch. (2025) 487:275–86. doi: 10.1007/s00428-025-04189-0 PMC12391220 40748381

[37] Chan KCA Woo JKS King A Zee BCY Lam WKJ Chan SL . Analysis of plasma Epstein-Barr virus DNA to screen for nasopharyngeal cancer. N Engl J Med. (2017) 377:513–22. doi: 10.1056/NEJMoa1701717 28792880

[38] Chan KCA Lam WKJ King A Lin VS Lee PPH Zee BCY . Plasma Epstein-Barr virus DNA and risk of future nasopharyngeal cancer. NEJM Evid. (2023) 2:EVIDoa2200309. doi: 10.1056/EVIDoa2200309, PMID: 38320164

[39] Li XQ Lin DF Cai YC Xie SH Lin KN Zhou HN . Diagnostic performance of EBV DNA load testing for nasopharyngeal carcinoma in nasopharyngeal swab outperforms the approach in other specimens. BMC Cancer. (2025) 25:1126. doi: 10.1186/s12885-025-14539-5, PMID: 40597842 PMC12211123

[40] Chen W Xia C Zheng R Zhou M Lin C Zeng H . Disparities by province, age, and sex in site-specific cancer burden attributable to 23 potentially modifiable risk factors in China: a comparative risk assessment. Lancet Glob Health. (2019) 7:e257–e69. doi: 10.1016/S2214-109X(18)30488-1, PMID: 30683243

