# Supplementary figures and images for: Extranodal nasal–orbital communicating lesions NK/T cell lymphoma with ocular symptoms as the initial manifestation misdiagnosed as sinusitis and orbital cellulitis: a case report and literature review
Source: Front Oncol. 2026 Apr 1;16:1732788. doi: 10.3389/fonc.2026.1732788 (PMC13079134; doi:10.3389/fonc.2026.1732788)

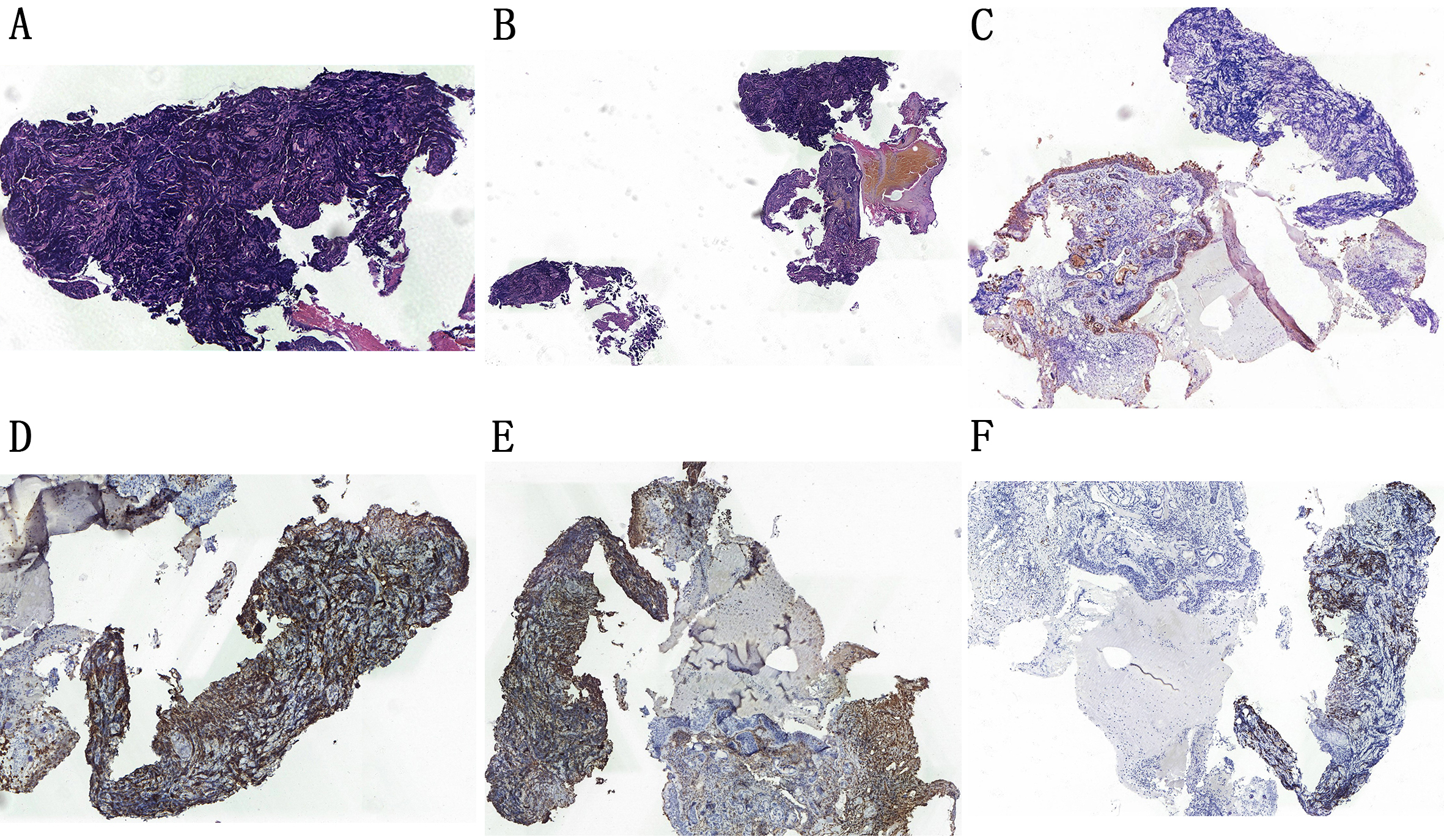

Supplement: Supplementary Figure 1 — Representative histopathologic and immunohistochemical findings in a patient with ENKTL. (A) H&E stain, ×100: Squeezed blue-stained unstructured diffuse cells. (B) H&E stain, ×40: Squeezed blue-stained unstructured diffuse cells. (C) CK, ×40. (D) LCA, ×200. (E) Vim, ×100. (F) Ki67, ×100. [file Image1.jpeg]

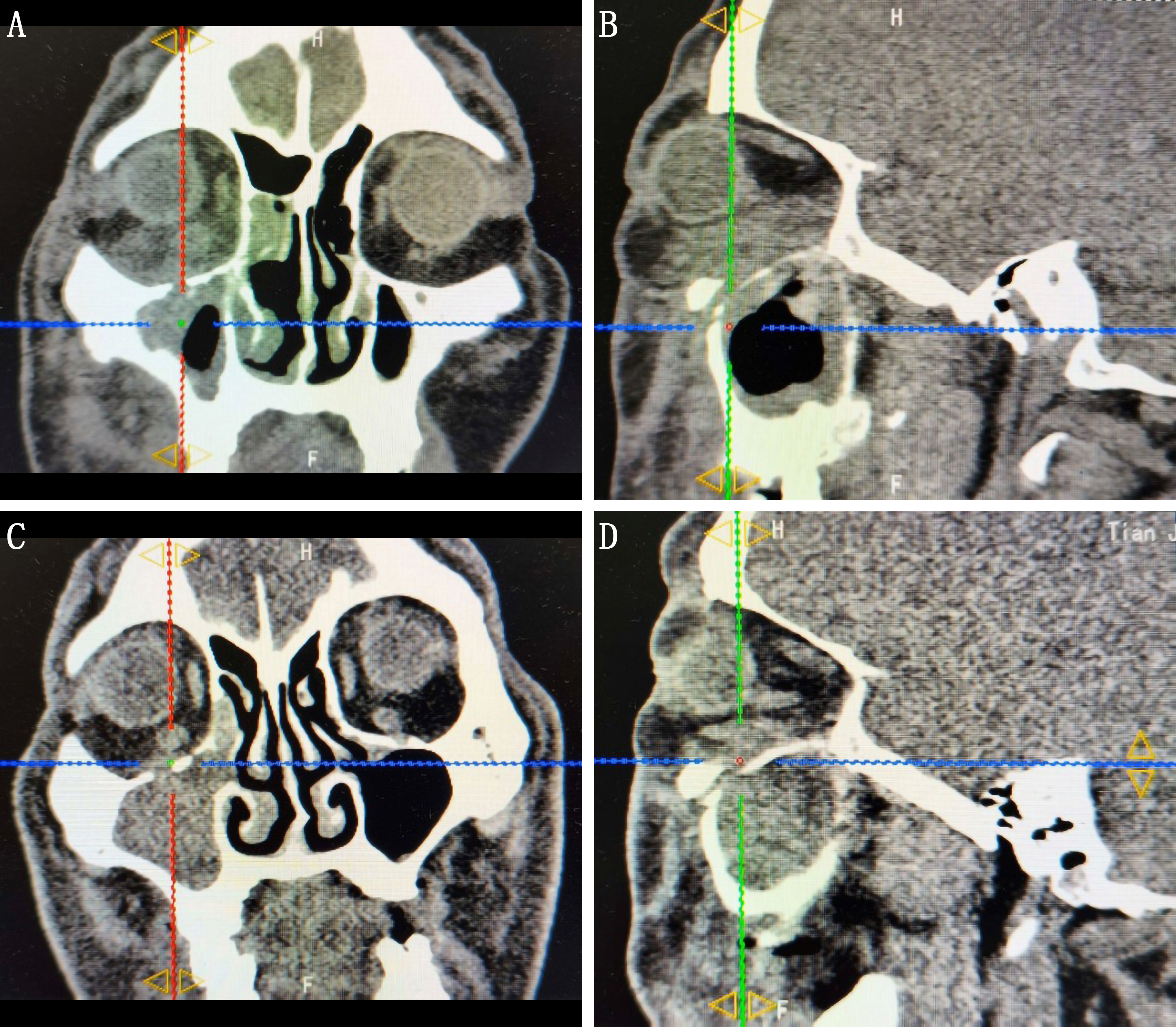

Supplement: Supplementary Figure 2 — (A, B) The area of the right inferior rectus muscle below the eyeball after the FESS surgery. (A) The coronal view. (B) The sagittal view. (C, D) The area of the right inferior rectus muscle below the eyeball before the FESS surgery. (C) The coronal view. (D) The sagittal view. [file Image2.jpeg]

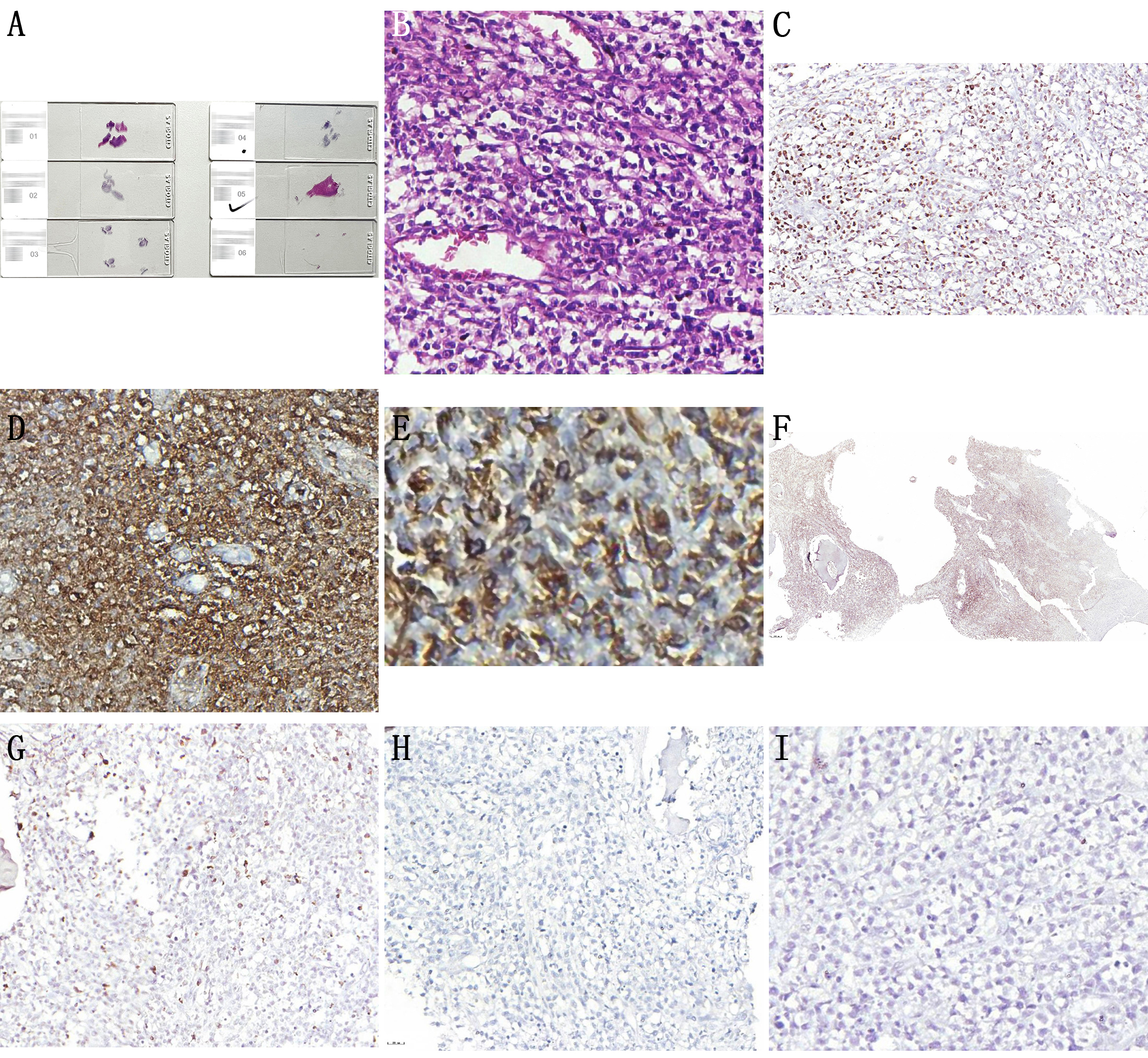

Supplement: Supplementary Figure 3 — Representative histopathologic and immunohistochemical findings in a patient with ENKTL. (A) Samplings from 6 sites (1. Inferior part of the right orbit 2. Right orbital bone 3. Inferior bone of the right orbit 4. Right infraorbital 5. Inside the right orbit 6. Right maxillary sinus). (B) H&E stain, ×200. (C) EBER ×200. (D) LCA, ×400. (E) VIM, ×400. (F) CD99, ×200. (G) Ki67, ×200. (H) CK, ×200. (I) CD20, × 200. [file Image3.jpg]
