# Supplementary material for: Extranodal nasal–orbital communicating lesions NK/T cell lymphoma with ocular symptoms as the initial manifestation misdiagnosed as sinusitis and orbital cellulitis: a case report and literature review
Source: Front Oncol. 2026 Apr 1;16:1732788. doi: 10.3389/fonc.2026.1732788 (PMC13079134; doi:10.3389/fonc.2026.1732788)
Supplement: Supplementary file 4 [file Table1.docx]

Supplemental Table 1. Slit lamp examination

| Category | OD | | | OS | | |
| --- | --- | --- | --- | --- | --- | --- |
| Date | 14th April | 11th May | 27th May | 14th April | 11th May | 27th May |
| Unaided visual acuity/Decimal | 0.8 | 0.6 | Hand motion | 0.6 | 0.6 | 0.6 |
| Intraocular pressure/mmHg | 14 | 19 | 19 | 19 | 17 | 17 |
| Eyelid | Edema,No tenderness | Congestion and edema, without obvious tenderness | Significant swelling, difficulty opening the eyes | Normal | Normal | Normal |
| Conjunctiva | Mild congestion | Congestion and edema | Congestion and edema | Mild congestion | Mild congestion | Mild congestion |
| Cornea | Transparent | Transparent | Transparent | Transparent | Transparent | Transparent |
| Anterior chamber | Flare(-) | Flare(-) | Flare(-) | Flare(-) | Flare(-) | Flare(-) |
| Pupils | Circle, 3mm,Light reflection (+) | Circle, 3mm,Light reflection (+) | Circle, 5mm,Light reflection (Slow to react) | Circle, 3mm,Light reflection (+) | Circle, 3mm,Light reflection (+) | Circle, 3mm,Light reflection (+) |
| Lenses | Crystal Light Mix | Crystal Light Mix | Crystal Light Mix | Crystal Light Mix | Crystal Light Mix | Crystal Light Mix |
| Fundus | The optic disc has a clear margin, and multiple cotton wool spots are visible on the retina. | The optic disc has a clear margin, and multiple cotton wool spots are visible on the retina. | The optic disc has a clear margin. Scattered cotton wool spots and small areas of hemorrhage visible on the retina | The optic disc has a clear margin, and multiple cotton wool spots are visible on the retina. | The optic disc has a clear margin, and multiple cotton wool spots are visible on the retina. | The optic disc has a clear margin, and multiple cotton wool spots are visible on the retina. |
| Eye movement | Downward restricted | The downward transfer restriction has worsened compared to last time | Partially restricted | Normal | Normal | Normal |
